# Cigarette Smoke Extract Exposure: Effects on the Interactions between Titanium Surface and Osteoblasts

**DOI:** 10.1155/2019/8759568

**Published:** 2019-04-22

**Authors:** Jie Yang, Shui-yi Shao, Wan-qing Chen, Chao Chen, Song-mei Zhang, Jing Qiu

**Affiliations:** ^1^Department of Oral Implantology, Affiliated Hospital of Stomatology, Nanjing Medical University, Nanjing 210029, China; ^2^Jiangsu Key Laboratory of Oral Disease, Nanjing Medical University, Nanjing 210029, China; ^3^Department of Oral Implantology, Huaxia Hospital of Stomatology, Suzhou, China; ^4^Department of General Dentistry, University of Rochester Eastman Institute for Oral Health, Rochester, NY, USA

## Abstract

The aim of this study was to explore the changes in the characteristics of titanium surface and the osteoblast-titanium interactions under cigarette smoke extract (CSE) exposure. In this study, CSE was used to simulate the oral liquid environment around the implant under cigarette smoke exposure. Titanium samples were immersed in CSE to explore the changes in the characteristics of titanium surface. The physical properties of titanium surface were measured, including surface micromorphology, surface elemental composition, roughness, and surface hydrophilicity. MC3T3-E1 cells were cultured on the titanium surface* in vitro* under different concentrations of CSE exposure, and cell adhesion, cell proliferation, and osteogenic differentiation were observed. The surface micromorphology and elemental composition of titanium surface changed under CSE exposure. No obvious changes were found in the surface roughness and the hydrophilicity of titanium samples. Moreover, the results of* in vitro* study showed that CSE exposure downregulated the cell spreading, proliferation, and osteogenic differentiation of MC3T3-E1 cells on the titanium surface. It could be speculated that some carbon-containing compounds from CSE adsorbed on the titanium surface and the osteoblast-titanium interactions were influenced under CSE exposure. It is hoped that these results could provide valuable information for further studies on smoking-mediated inhibition of implants osseointegration.

## 1. Introduction

Smoking is common in modern civilization. The estimated prevalence of daily tobacco smoking among the adult population was 15.2% [1]. Tobacco is the single leading and preventable cause of death worldwide [2]. Smoking is related to several systemic diseases [3–5]. It has also been regarded as a major risk factor that triggers the development of oral diseases [6]. As is well known, despite high success rate, dental implant failures occur occasionally. Smoking is considered as one of the major risk factors for the failure of dental implants [7–10]. Clinical studies revealed that smoking could affect initial implant survival rates and early osseointegration [11–14]. In fact, cigarette smoke is a highly dynamic complex consisting of more than 3800 compounds, including oxidants, nicotine, heavy metals, aromatic hydrocarbons, and aldehydes [15, 16]. Although the components of cigarette smoke have been identified, the mechanism of its effect on the osseointegration between bone and dental implant is still unclear.

In recent years, dental implant technology has been developed continuously [17, 18]. Dental rehabilitation of partially or totally edentulous patients with dental implants has become a common practice in the past decades [19]. Dental implants play an important role in the dental restoration [20]. Titanium is commonly used for dental implants because of its excellent biocompatibility and satisfactory mechanical properties. Osseointegration is crucial to the dental implant technology [21, 22]. Osseointegration is defined as “the firm, direct, and lasting biological attachment of a metallic implant to vital bone without intervening connective tissue” by the American Academy of Implant Dentistry. Dental implants fail mostly because of an incomplete establishment of the bone-implant interface [23]. From a biochemical perspective, it is well accepted that hydrocarbons from the atmosphere, water, or cleaning solution could adsorb on the titanium surfaces [24]. A previous study reported that osteoblast attachment to titanium surfaces was inversely correlated with the hydrocarbon adsorption on titanium surfaces [25]. It provides us with a new way of thinking that substances adsorption on the titanium surface may account for the failure of osseointegration.

Could the substances of cigarette smoke adsorb on the implant surface? Some studies reported that implants were exposed to the oral cavity in the early process of osseointegration [26–29]. In addition, smoking could inhibit the healing of gingiva and the function of gingival fibroblasts [20, 30] and even increased the incidence of implant exposure [7, 9]. In the case of nonsubmerged healing, the interface between implant and bone is directly exposed to the oral cavity. In the case of submerged healing, spontaneous implant exposure does exist and could account for crestal bone loss around the dental implant during the early osseointegration phase [31]. Tal* et al*. reported that the spontaneous early exposure rate of submerged implants could reach as high as 13.7% [26]. Thus, substances of cigarette smoke might adsorb on the implant through the exposure of dental implants in the oral cavity. It could be speculated that cigarette smoke could affect the early osseointegration of the implant through its exposure.

Up to date, the exact mechanism underlying the smoking-mediated inhibition of the osseointegration remains to be elucidated. To the best of the authors' knowledge, little attention has been paid to the influence of the cigarette smoke on both titanium implant surface and osteoblast behavior. We hypothesized that cigarette smoke exposure could influence the interaction between titanium surface and osteoblast. In the present study, preliminary model for investigating the effects of cigarette smoke on the osseointegration of titanium implants was provided. Cigarette smoke extract (CSE) was commonly used to simulate smoking in the study of pulmonary diseases* in vitro *[32], culturing human lung fibroblasts [33], pulmonary epithelial cell [34], and primary nasal epithelial cells [35]. In this work, CSE is used to simulate the oral liquid environment around the implant under cigarette smoke exposure [36]. We sought to evaluate the effect of CSE exposure on the characteristics of titanium surface and the role of CSE exposure on osteoblast-titanium interactions. We hope that the results would provide valuable information for exploring the initiating causes of the smoking-mediated inhibition of the osseointegration.

## 2. Materials and Methods

### 2.1. CSE Preparation

**Figure 1:**
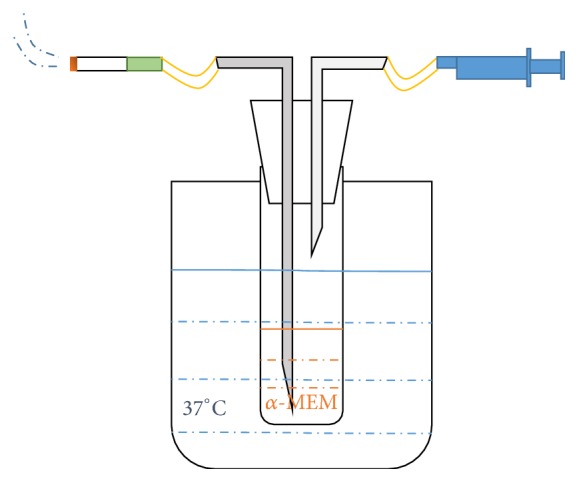
Schematic representation of the CSE-generating machine. CSE was freshly prepared by bubbling the smoke drawn from a commercially available cigarette through 10 mL prewarmed (37°C) cell culture medium. The cigarette was smoked at a rate of 50 mL over a period of 2 s, followed by a 28 s pause.

As shown in Figure 1, CSE was freshly prepared by bubbling the smoke drawn from a lit cigarette (nicotine 0.8 mg, tar 11 mg, Marlboro, China) through 10 mL prewarmed (37°C) cell culture medium (*α*-MEM, Gibco, USA) [37]. The cigarette was smoked at a rate of 50 mL over a period of 2 s, followed by a 28 s pause, matching the smoking habits of an average smoker [38]. The obtained extract was filtered through a 0.22 *μ*m pore filter (Millipore, Bedford, MA, USA). The concentration of the resulting undiluted extract was assigned a value of 1. Subsequently, it was diluted with cell culture medium (*α*-MEM, Gibco, USA) and the serial diluted extracts were assigned relative values of 10%, 5%, or 2% (10-, 20-, or 50-fold diluted extract), respectively [37]. 0% CSE was designated as cell culture medium (*α*-MEM, Gibco, USA). All the extracts contained 10% fetal bovine serum (FBS, Gibco, USA) and 1% penicillin/streptomycin (Gibco, USA). The extracts were freshly prepared before experiments.

### 2.2. Sample Preparation

Pure titanium (99 wt.% purity, China) samples were custom-made as disks (Ø = 5, 30 mm). The samples were ground using a series of silicon carbide (SiC) papers (600, 800, 1200, and 1500 grit). Subsequently, the samples were cleaned in an ultrasonic bath and dried in the ambient atmosphere. Then, the samples were immersed in 0%, 2%, 5%, and 10% CSE in a humidified atmosphere containing 5% CO_2_ and 95% air at 37°C. The samples immersed in 0% CSE were denoted as Ti-control and used as the control group, while those immersed in 2%, 5%, and 10% CSE were denoted as Ti-CSE-2, Ti-CSE-5, and Ti-CSE-10, respectively, and used as experimental groups.

### 2.3. Surface Characterization

Titanium samples with a diameter of 0.5 cm were immersed in 0.1 mL CSE (0%, 2%, 5%, and 10%) at 37°C in a humidified atmosphere of 5% CO_2_ and 95% air. After 3 d, the samples were rinsed by double-distilled water and then dried before being used. Field-emission scanning electron microscopy (SEM, LEO, Germany) was employed to characterize the surface morphology of the prepared samples. The X-ray photoelectron spectroscopy (XPS) data were obtained with a Thermo Scientific Escalab 250Xi spectrometer. The chemical states of the components were also detected. The spectra were calibrated with respect to the C 1s signal at 284.8 eV [39]. The roughness and three-dimensional views of the surfaces of Ti-control, Ti-CSE-2, Ti-CSE-5, and Ti-CSE-10 samples were evaluated by confocal three-dimensional surface topography instrument (UP series, RTEC, USA). The surface hydrophilicity of Ti-control, Ti-CSE-2, Ti-CSE-5, and Ti-CSE-10 samples was measured by the contact angle of 2 *μ*L H_2_O using a contact angle measurement system (Model SL200B, Solon, China). A total of 16 titanium samples (Ø = 5 mm) were used for the surface morphology, which were equally distributed among 4 group. The number and grouping of samples used for roughness/three-dimensional view and hydrophilicity are the same as the surface morphology. Ti-control and Ti-CSE-10 were selected as representative samples for determining the surface elemental compositions, and 8 titanium samples (Ø = 5 mm) were used for it.

### 2.4. Cell Culture

MC3T3-E1 cell is a highly characterized murine preosteoblastic cell line established from mouse calvaria [40]. MC3T3-E1 cell used in this study was purchased from Shanghai Cell Bank of Chinese Academy of Sciences (Shanghai, China). The cells were cultured in *α*-Minimum Essential Medium (*α*-MEM, Gibco, USA) supplemented with 10% fetal bovine serum (FBS, Gibco, USA) and 1% antibodies containing penicillin/streptomycin (Gibco, USA) at 37°C in a humidified atmosphere of 5% CO_2_ and 95% air. The culture medium was changed once every 2 d. When the cells reached 80% confluency, they were trypsinized and passaged at a ratio of 1:4.

### 2.5. Cell Adhesion and Spreading Assay

To observe the morphology of MC3T3-E1 cells in different groups, the cells were seeded onto titanium samples with 0.5 cm diameter in 96-well plates (5×10^3^ cells/well). The wells were filled with 0.1 mL 0, 2, 5, and 10% CSE in different groups. After 4 h and 8 h of incubation, the cells on the titanium samples were fixed with 4% paraformaldehyde (PAF) for 10 min at room temperature. After permeabilization with 0.5% Triton X-100, the cells in different groups were stained with 100nM rhodamine-phalloidin (Cytoskeleton, USA) at room temperature for 30 min in the dark. Then, cells were stained again with 100nM 4',6'-diamidino-2-phenylindole (DAPI) (Beyotime, China) for 30 s. The images of adherent MC3T3-E1 cells in four different groups were acquired at 200× magnification with a laser scanning confocal microscopy (LSM710, Zeiss, Germany). A total of 32 titanium samples (*Ø* = 5 mm) were used in this assay and equally distributed among each group. Specifically, 4 groups at 2 time points were included in this assay, and the center field of each specimen was observed.

### 2.6. Cell Proliferation Assay

Cell proliferation ability was assessed by the Cell Counting Kit-8 (CCK-8, Biosharp, China). Briefly, MC3T3-E1 cells were seeded onto titanium samples with 0.5 cm diameter in 96-well plates (2×10^3^ cells/well). The wells were filled with 0.1 mL 0, 2, 5, and 10% CSE in different groups. After 1 d, 3 d, and 6 d of incubation, each well was refilled with a fresh solution consisting of 10 *μ*l CCK-8 solution (Biosharp, China) and 0.1 mL culture medium, followed by 2 h incubation at 37°C. Subsequently, the absorbance of the incubated solutions at 450 nm wavelength was measured by a microplate reader (Spectramax190, MD, USA). A total of 48 titanium samples (Ø = 5 mm) were used in this assay and equally distributed among each group. Specifically, 4 groups at 3 time points were included in this assay, and each group included four duplicate wells.

### 2.7. Alkaline Phosphatase (ALP) Activity Assay

For the ALP activity assay, MC3T3-E1 cells were seeded onto titanium samples with 3 cm diameter in 6-well plates (2×10^5^ cells/well). The wells were filled with 2.5 mL 0, 2, 5, and 10% CSE in different groups. After 7 d and 14 d of incubation, the cells on the samples were rinsed with PBS and then lysed (4°C, 30 min) using radio-immunoprecipitation assay (RIPA) buffer (Leagene, China) in the presence of 1 mM phenylmethylsulfonyl fluoride (PMSF, Leagene, China). The collected lysates were then centrifugated at 12000 rpm at 4°C for 10 min. The protein concentration of the liquid supernatants was determined using a BCA protein assay kit (Keygen Biotech, China). Then, the ALP activity was assessed using an AKP assay kit (Jiancheng Bioengineering Institute, China). A total of 32 titanium samples (Ø = 30 mm) were used in this assay and equally distributed among each group. Specifically, 4 groups at 2 time points were included in this assay, and each group included four duplicate wells.

### 2.8. Western Blotting

Run-related transcription factor 2 (Runx2) and osterix (OSX) protein expressions in different groups were examined by Western blotting analyses. MC3T3-E1 cells were seeded onto titanium samples with 3 cm diameter in 6-well plates (2×10^5^ cells/well). The wells were filled with 2.5 mL 0, 2, 5, and 10% CSE in different groups. After 7 d and 14 d of incubation, cells on the samples were gently rinsed with PBS before lysis in RIPA buffer in the presence of 1 mM PMSF. The protein concentration of the liquid supernatants was determined using a BCA protein assay kit. Proteins were separated by SDS-PAGE and transferred to the polyvinylidene fluoride (PVDF) membranes (Millipore, USA). The membranes were then blocked with 5% skim milk for 1 h at room temperature. The blocked membranes were washed with TBS-T buffer and probed with different primary rabbit antibodies against Runx2 (12556, CST, USA), OSX (22552, Abcam, UK) and GAPDH (100118, GeneTex, USA) overnight at 4°C. Subsequently, PVDF membranes were incubated with a secondary antibody (anti-rabbit, ZB-2301, ZSGB-BIO, China) for 2 hours. The immunoreactive proteins were detected by using Immobilon Western Chemiluminescent HRP substrate (Millipore, USA). The relative expression levels of Runx2 and OSX proteins were normalized to GAPDH. A total of 24 titanium samples (Ø = 30 mm) were used in this assay and equally distributed among each group. Specifically, 4 groups at 2 time points were included in this assay, which was performed in triplicate.

### 2.9. Statistical Analysis

Statistical analysis was performed using SPSS 22.0 (SPSS Inc., Chicago, IL, USA). The data were expressed as mean ± standard deviation and analyzed by one-way analysis of variance (ANOVA). Statistical significance level was set at* P* < 0.05.

## 3. Results and Discussion

### 3.1. Surface Micromorphology and Elemental Composition

**Figure 2:**
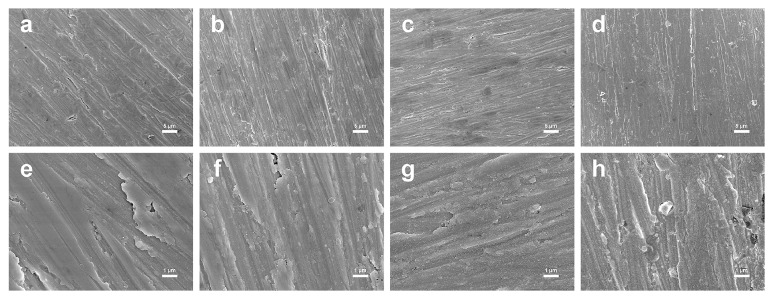
Scanning electron microscopy images of titanium samples under CSE exposure for 3 d. Upper panel ((a) Ti-control; (b) Ti-CSE-2; (c) Ti-CSE-5; (d) Ti-CSE-10) images at 2000× magnification. Lower panel ((e) Ti-control; (f) Ti-CSE-2; (g) Ti-CSE-5; (h) Ti-CSE-10) images at 10000× magnification.

The result of SEM observation was shown in Figure 2. The surface morphologies of Ti-control, Ti-CSE-2, Ti-CSE-5, and Ti-CSE-10 samples did not show obvious differences under low magnification (Figures 2(a), 2(b), 2(c), and 2(d)). Different from the surfaces of Ti-control samples, particles could be observed on the surfaces of Ti-CSE-2, Ti-CSE-5, and Ti-CSE-10 samples under higher magnification (Figures 2(e), 2(f), 2(g), and 2(h)). From Ti-CSE-2 to Ti-CSE-10, an increasing number of particles appeared on the sample surfaces (Figures 2(f), 2(g), and 2(h)).

During the process of osseointegration, the formation and maintenance of new bone at the implant surfaces ensure the success of dental implants [41]. In this study, from the results of SEM observation, particles were found on the titanium surfaces under CSE exposure (Figure 2). Previous studies showed that CSE contained thousands of chemicals [15, 16]. We speculated that these particles were derived from CSE. It could be conjectured that these substances adsorbed on the titanium surface, thereby influencing the surface micromorphology of the titanium surface.

**Figure 3:**
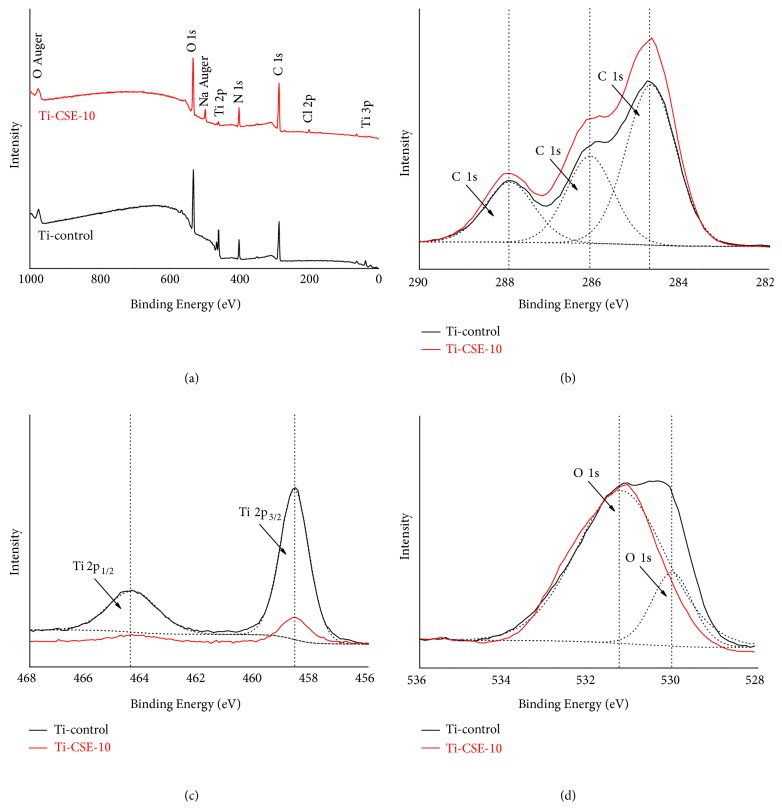
XPS spectra of titanium samples ((a) survey spectra of the samples; (b) high-resolution spectra of C 1 s on the samples; (c) high-resolution spectra of Ti 2p on the samples; (d) high-resolution spectra of O 1s on the samples).

The survey spectra by XPS acquired from the Ti-control and Ti-CSE-10 samples were displayed in Figure 3. Titanium (Ti), oxygen (O), carbon (C), and nitrogen (N) were present on both Ti-control and Ti-CSE-10 samples, while sodium (Na) and chlorine (Cl) only appeared on Ti-CSE-10 samples (Figure 3(a)). In Figure 3(b), the C 1s high-resolution spectra exhibited three peaks at about 284.68 eV, 286.08 eV, and 287.98 eV, respectively. High-energy peaks of C 1s were detected on Ti-control samples, which could probably be attributed to the immersion in the cell medium. Notably, high-energy peaks of C 1s were detected on Ti-CSE-10 samples. Therefore, some carbon-containing compounds from CSE adsorbed on Ti-CSE-10 samples. However, lower-energy peaks of Ti 2p were detected at 464.38 eV and 458.58 eV on Ti-CSE-10 samples compared to the Ti-control samples (Figure 3(c)). Moreover, the O 1s peaks at 529.98 eV, indicating the existence of titanium dioxide (TiO_2_), appeared on Ti-control samples rather than the Ti-CSE-10 samples (Figure 3(d)).

From the XPS data, sodium (Na) and chlorine (Cl) appeared on the Ti-CSE-10 samples (Figure 3(a)) and high-energy peaks of C 1s were detected on the Ti-CSE-10 samples rather than Ti-control samples (Figure 3(b)), consistent with the result of SEM. Moreover, high-energy peaks of Ti 2p were detected on the Ti-control samples (Figure 3(c)), and the O 1s peaks at 529.98 eV (Figure 3(d)), indicating the existence of titanium dioxide (TiO_2_), appeared on the Ti-control samples rather than the Ti-CSE-10 samples. This comparison of results suggested that carbon-containing compounds adsorbed on the Ti-CSE-10 samples and the TiO_2_ layer of the Ti-CSE-10 samples was covered after the immersion in the CSE. We speculated that the carbon-containing compounds adsorbing on the titanium surface in the smokers might affect the ossteointegration of implants. This result was consistent with a previous study by Att* et al.* [25], reporting progressive accumulation of hydrocarbons on titanium implants with passage of time. The C1s peak was considerably higher on the surface after 4 weeks of storage in dark ambient conditions than on the new surface. Normally, hydrocarbons contamination was present on titanium implants used for clinical and experimental use, which were routinely stored in ambient conditions [42, 43]. Along this line, carbon-containing compounds from CSE could adsorb on titanium surface, as found in the present study.

### 3.2. Surface Roughness and the Hydrophilicity

**Figure 4:**
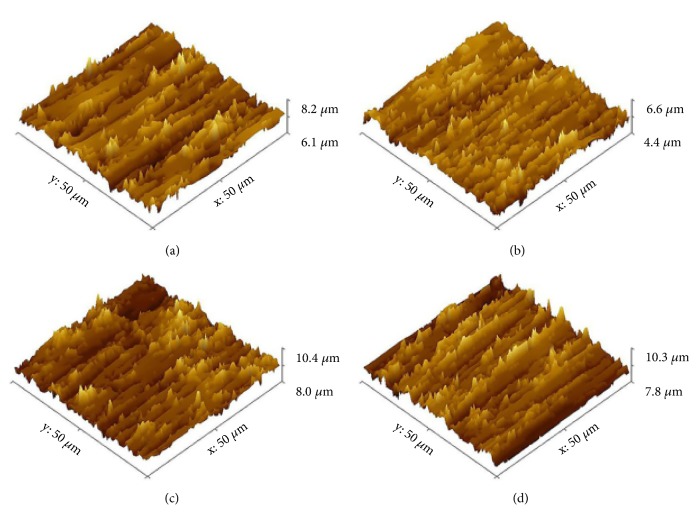
Three-dimensional morphology of Ti-control (a), Ti-CSE-2 (b), Ti-CSE-5 (c), and Ti-CSE-10 (d) samples.

**Figure 5:**
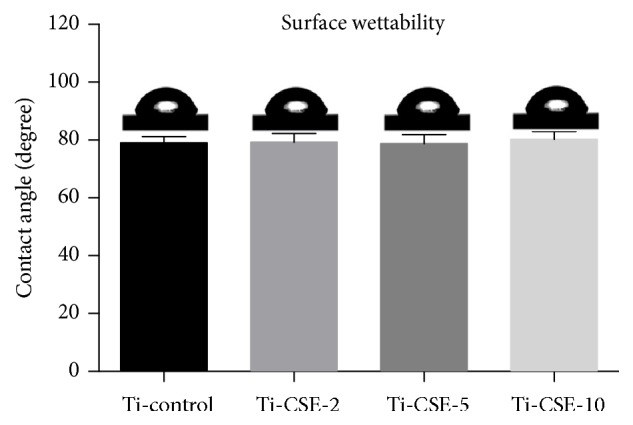
The contact angles of Ti-control, Ti-CSE-2, Ti-CSE-5, and Ti-CSE-10 samples.

The results of surface roughness assay were shown in Table 1. From the results, Ti-control, Ti-CSE-2, Ti-CSE-5, and Ti-CSE-10 samples revealed similar surface roughness values (R_a_). Besides, the three-dimensional views of Ti-control, Ti-CSE-2, Ti-CSE-5, and Ti-CSE-10 samples did not show any obvious differences (Figure 4). As shown in Figure 5, the wettability assay did not reveal any significant differences of contact angles among Ti-control, Ti-CSE-2, Ti-CSE-5, and Ti-CSE-10 samples. These results showed that CSE exposure did not influence the roughness or the hydrophilicity of the titanium surfaces.

Thus, the physical properties of titanium samples under CSE exposure revealed that CSE could alter the micromorphology and elemental composition of titanium surface, excluding surface roughness and hydrophilicity. Furthermore, a study by Aita* et al.* [44] explored inverse correlation between carbon element on titanium and its osteoblast attractiveness. It suggested that the rate of osteoblast attachment increased exponentially with the progressive decrease of carbon. As reported by Att* et al.* [25], the amount of osteogenic cells adhering to the titanium implant surface decreased with the increase of hydrocarbons on the titanium surface.

### 3.3. Adhesion and Proliferation Ability of Osteoblasts under CSE Exposure

**Figure 6:**
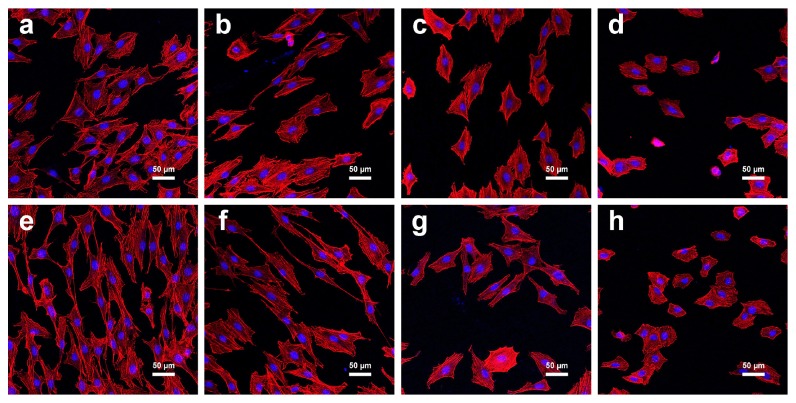
Fluorescence images of MC3T3-E1 cells spreading in four groups after 4 h and 8 h of incubation (magnification 200×). Upper panel ((a) Ti-control; (b) Ti-CSE-2; (c) Ti-CSE-5; (d) Ti-CSE-10) displays cell spreading after 4 h of incubation. Lower panel ((e) Ti-control; (f) Ti-CSE-2; (g) Ti-CSE-5; (h) Ti-CSE-10) displays cell spreading after 8 h of incubation.

The adhesion behavior of MC3T3-E1 cells was observed under laser scanning confocal microscopy (Figure 6). After incubation for 4 h and 8 h, the differences in the cell morphology were observed among different groups. With the increase of CSE concentration, the cells extended fewer pseudopodia and spread less. The cells in Ti-CSE-10 group demonstrated the worst cell-adhesion ability, followed by the Ti-CSE-5 and then the Ti-CSE-2 groups. On the contrary, the cells in Ti-control group exibihited relatively normal morphology.

**Figure 7:**
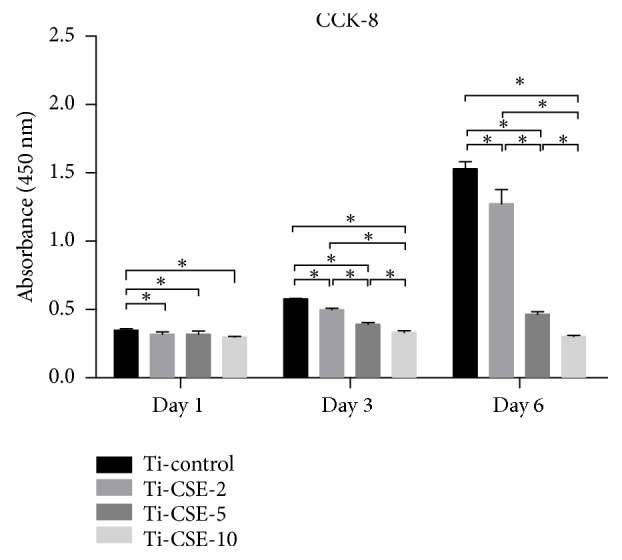
After incubation for 1, 3, and 6 d, cell proliferation ability of MC3T3-E1 cells adhering to the titanium samples in Ti-control, Ti-CSE-2, Ti-CSE-5, and Ti-CSE-10 groups was analyzed by the CCK-8 assay. *∗* indicated significant differences between different samples (*P* < 0.05).

The cell proliferation ability was measured by CCK-8 assay (Figure 7). MC3T3-E1 cells steadily proliferated in all groups during culture period, except the Ti-CSE-10 groups. After 3 and 6 d of incubation, in Ti-control group, the cells proliferated and survived significantly more than those in Ti-CSE-2, Ti-CSE-5, and Ti-CSE-10 groups. CSE exposure downregulated the proliferation of MC3T3-E1 cells in a dose-dependent fashion.

The results of cell spreading and proliferation showed that the CSE exposure influenced the biological behavior of the osteoblasts on the titanium samples. In addition, a high concentration of CSE exhibited cellular cytotoxicity. This evidence revealed that the CSE exposure could affect the adhesion and proliferation of the osteoblasts on the titanium surface. This was in consistency with a recent study by Cyprus* et al*. [45] who employed CSE exposure as a stimulating factor on macrophages and MSCs and found that CSE exposure attenuated cell viability of macrophage and MSC in a dose-dependent manner. It demonstrated that CSE exposure decreased osteogenic differentiation and anti-inflammatory interleukin production while increasing proinflammatory interleukin production in macrophages and MSCs on titanium surfaces. Reportedly, the adhesion and subsequent proliferation of osteoblastic cells on the implant surface are responsible for implant osseointegration [46]. CSE exposure could inhibit chemotaxis mediated by human bone marrow osteoprogenitor cells and osteoblast-like cells [47]. Acetaldehyde, one of the components of CSE, also inhibited the cell proliferation in human osteoblastic cells [46]. Thus, the conclusions of these previous studies confirmed the current results in the present study.

### 3.4. Osteogenic Differentiation Behavior of Osteoblasts under CSE Exposure

**Figure 8:**
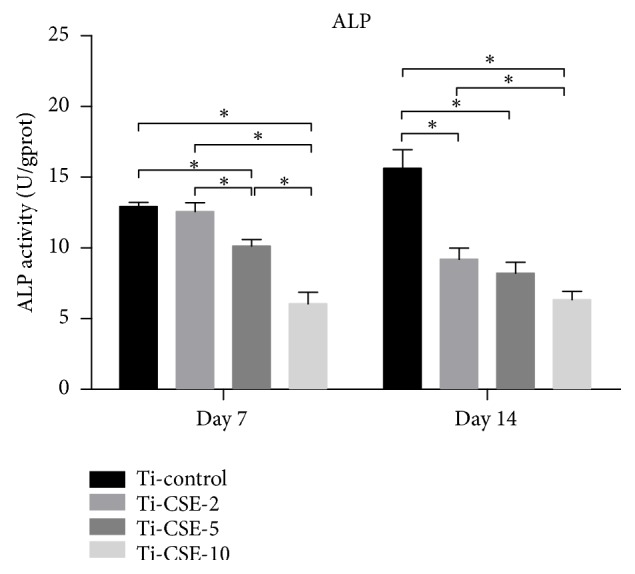
The alkaline phosphatase (ALP) activity of MC3T3-E1 cells in Ti-control, Ti-CSE-2, Ti-CSE-5, and Ti-CSE-10 groups after incubation for 7 d and 14 d. *∗* indicated significant differences between different samples (*P* < 0.05).

The results of ALP activity assay were shown in Figure 8. After incubation for 7 d, the MC3T3-E1 cells in Ti-control group showed higher ALP activity than those in the Ti-CSE-2, Ti-CSE-5, and Ti-CSE-10 groups. After incubation for 14 d, the ALP activity of MC3T3-E1 cells in Ti-control group showed a clear dominance compared to those in the Ti-CSE-2, Ti-CSE-5, and Ti-CSE-10 groups. What is more, the ALP activity of osteoblasts in the Ti-control group had a significant increase at the end of culture. Interestingly, the ALP activity of osteoblasts in the Ti-CSE-2, Ti-CSE-5, and Ti-CSE-10 groups decreased with the passage of culture time.

**Figure 9:**
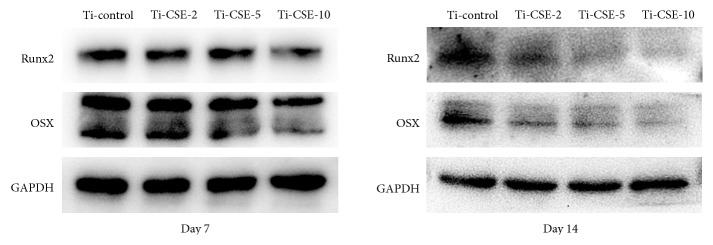
Osteogenic-related protein expression levels of Runx2 and OSX of MC3T3-E1 cells in Ti-control, Ti-CSE-2, Ti-CSE-5, and Ti-CSE-10 groups were detected by Western blotting after 7 d and 14 d of incubation.

The osteogenic lineage protein expression levels in the four groups were examined by Western blotting analysis. As shown in Figure 9, at culture days 7 and 14, the expression level of Runx2 gradually declined with the increase of CSE concentration. MC3T3-E1 cells in three experimental groups exhibited lower expression levels of Runx2 than those in Ti-control group. Similar to Runx2, the expression level of OSX steadily declined with the increase of CSE concentration at culture days 7 and 14. The Ti-CSE-10 group exhibited lowest expression levels of OSX. Hence, the CSE exposure could negatively affect the cell differentiation activities of the osteoblasts.

Osseointegration and osteogenic differentiation are critical for clinical outcomes involving implants in the orthopedics and dentistry [48–50]. ALP is considered as an early marker for osteogenic differentiation [51]. In this study, the ALP activity of osteoblasts on the titanium surface significantly decreased under the CSE exposure. This result was in agreement with the observations of Cyprus* et al.* [45], reporting that CSE exposure downregulated alkaline phosphatase activity of MSCs. Runx2 and Osterix (OSX) were commonly selected to analyze the osteogenic differentiation abilities. Runx2, a vital transcription factor, was expressed during the early stage of osteogenic differentiation and directs mesenchymal progenitor cells to preosteoblasts. OSX is required for the differentiation of preosteoblasts into mature osteoblasts [52]. In this study, the expression level of Runx2 and OSX steadily declined with the increase of CSE concentration. Previous studies found that CSE exposure exerts a negative impact on the osteogenic differentiation [53, 54]. The conclusions of these studies were consistent with our results. Therefore, it can be speculated that these CSE-originated carbon-containing compounds, which adsorbed on the titanium surface, affected osteogenic differentiation ability of osteoblasts.

Several* in vitro* studies have demonstrated that specific components of cigarette smoke, like nicotine, acrolein, and acetaldehyde, inhibited functions of human bone marrow-derived cells or osteoblastic cells cultured on titanium surfaces [46, 55]. However, those studies focused on single or several compounds of cigarette smoke. Another method for studying tobacco smoke* in vitro* was to prepare the cigarette smoke extracts. Since the usage of CSE is limited by the quality of the smoke extract preparation, we elected to use a smoke extract preparation method provided by Bernhard* et al.* [38], which guaranteed a similarity to the* in vivo* situation. According to the method, we controlled the puffing rhythm to mimic the average smoker, since the actual tobacco smoke toxicity was affected by the intensity, speed, and periodicity of the airflow through the cigarette [38]. With the help of this* in vitro* model, effect of cigarette smoke on implants can be explored at the biomolecular level. It should be mentioned that differences exist between the* in vitro* conditions and the actual* in vivo* exposure at the bone-implant interface. Therefore, further* in vivo* experiments with animals are required.

It was well understood that the predictable success of a dental implant was dependent on the level of crestal bone preservation around implant. A number of clinical researches reported that bone loss around implant was higher for smokers compared with nonsmokers. For instance, Moraschini* et al.* observed a statistically significant difference in marginal bone loss between the smoking group and the nonsmoking group, in favour of the nonsmoking group [56]. Vervaeke* et al.* reported that the estimated bone loss around implant was 1.2 mm higher for smokers compared with nonsmokers [57]. Furthermore, an* in vivo* study reported that cigarette smoke inhalation could result in a decreased bone healing around titanium implants [58]. While those published works have demonstrated that smoking is closely related to peri-implant bone loss, the exact mechanism did not reach a consensus. Through* in vitro *and* in vivo* studies using the method of CSE exposure, which was similar to our study, Cyprus* et al.* [45] attributed the deleterious effect of smoking on the osseointegration to the inflammatory response. According to this study, in parallel with our research, CSE exposure model may be useful for predicting how cigarette smoke may adversely affect the outcome of implants.

The present study explored the negative influence of CSE exposure on the cell-material interactions. It investigated the changes in the characteristics of titanium surface and biological responses of MC3T3-E1 cells to titanium surface under CSE exposure. The results uncovered that the CSE-originated carbon-containing compounds could adsorb on the titanium surface in the osteoblast-titanium interactions. We hope that the results of this study could be valuable for elucidating the exact effects of cigarette smoking on the early osseointegration of the titanium implant. In addition, the* in vivo* impacts of CSE exposure on the osteoblast-titanium interactions, as well as its underlying molecular mechanism, require further investigations.

## 4. Conclusions

In this study, we investigated effect of CSE exposure on the titanium surface characteristics and the osteoblast-titanium interactions. The surface micromorphology and elemental composition of titanium surface changed under CSE exposure. The analysis of surface characteristics showed that some carbon-containing compounds adsorbed on the titanium surface. Additionally, the results of* in vitro* study showed that CSE exposure downregulated the cell spreading, proliferation, and osteogenic differentiation of MC3T3-E1 cells on the titanium surface. These findings demonstrated that CSE exposure altered the micromorphology and elemental composition of titanium surface due to the carbon-containing compounds adsorption, which, in turn, influenced the osteoblast-titanium interactions. Therefore, we suggested that the carbon-containing compounds adsorption might be an important cause of smoking-mediated inhibition of the osseointegration. It is earnestly hoped that the present study could provide valuable information for exploring the initiating causes of smoking-mediated inhibition of the implant osseointegration.

## Figures and Tables

**Table 1:** R_a_ values of surface roughness of four different groups (n=4).

Group	R_a_ (*μ*M)
Ti-control	0.205 ± 0.016
Ti-CSE-2	0.186 ± 0.011
Ti-CSE-5	0.192 ± 0.008
Ti-CSE-10	0.198 ± 0.012

## Data Availability

The data used to support the findings of this study are available from the corresponding author upon request.
